# A novel and fully automatic spike-sorting implementation with variable number of features

**DOI:** 10.1152/jn.00339.2018

**Published:** 2018-07-11

**Authors:** Fernando J. Chaure, Hernan G. Rey, Rodrigo Quian Quiroga

**Affiliations:** ^1^Centre for Systems Neuroscience, University of Leicester, Leicester, United Kingdom; ^2^Instituto de Ingeniería Biomédica, UBA, Buenos Aires, Argentina; ^3^Estudios de Neurociencias y Sistemas Complejos (ENYS), CONICET - Hospital El Cruce - UNAJ, Florencio Varela, Argentina; ^4^Instituto de Biología Celular y Neurociencias “Prof. E. De Robertis”, Facultad de Medicina, UBA, Buenos Aires, Argentina

**Keywords:** neurophysiology, single-neuron recordings, spike sorting, tetrode

## Abstract

The most widely used spike-sorting algorithms are semiautomatic in practice, requiring manual tuning of the automatic solution to achieve good performance. In this work, we propose a new fully automatic spike-sorting algorithm that can capture multiple clusters of different sizes and densities. In addition, we introduce an improved feature selection method, by using a variable number of wavelet coefficients, based on the degree of non-Gaussianity of their distributions. We evaluated the performance of the proposed algorithm with real and simulated data. With real data from single-channel recordings, in ~95% of the cases the new algorithm replicated, in an unsupervised way, the solutions obtained by expert sorters, who manually optimized the solution of a previous semiautomatic algorithm. This was done while maintaining a low number of false positives. With simulated data from single-channel and tetrode recordings, the new algorithm was able to correctly detect many more neurons compared with previous implementations and also compared with recently introduced algorithms, while significantly reducing the number of false positives. In addition, the proposed algorithm showed good performance when tested with real tetrode recordings.

**NEW & NOTEWORTHY** We propose a new fully automatic spike-sorting algorithm, including several steps that allow the selection of multiple clusters of different sizes and densities. Moreover, it defines the dimensionality of the feature space in an unsupervised way. We evaluated the performance of the algorithm with real and simulated data, from both single-channel and tetrode recordings. The proposed algorithm was able to outperform manual sorting from experts and other recent unsupervised algorithms.

## INTRODUCTION

**Fig. 1.**
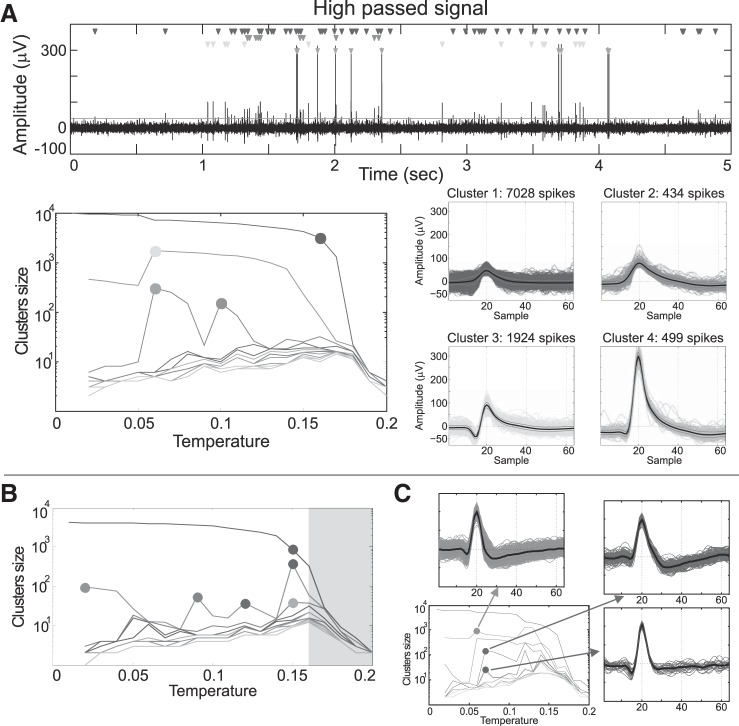
Improvements of the proposed method. *A*: example of an extracellular recording from the human right entorhinal cortex. *Top*: black trace shows the high-frequency content of the signal (between 300 and 3,000 Hz in this example). Neurons located more than ∼150 μm away from the tip of the electrode contribute to the background noise, so their spikes cannot be detected. Closer neurons (between 50 and 150 μm away from the tip of the electrode) generate spikes larger than the background noise, but they cannot be isolated into different units, thus being associated with the multiunit activity (cluster 1). Finally, nearby neurons (<50 μm) have even larger spikes, and sorting algorithms allow us to assign the recorded spikes to the putative neurons that generated them (clusters 2–4). Time of occurrence of each spike is marked with a triangle associated with the 4 isolated clusters. *Bottom*, *left*: temperature map shows all the different partitions generated by the superparamagnetic clustering. Each line is associated with the size of the *k*th cluster ranked by size at each temperature. The filled circles denote the location of the clusters depicted on the right, which have been selected by an expert user who manually optimized the solution of the former *Wave_clus*. *B*: example of the detection of the border of the superparamagnetic regime. *T_B_* was identified at *T* = 0.16, so the partitions at *T_i_ ≥ T_B_* were discarded (gray shaded area). *C*: example of the inclusion criterion where a cluster was split in 2 at a higher temperature. The overlap coefficient between the marked clusters at *T* = 0.07 and the marked cluster at *T* = 0.06 was equal to 1, so the clusters at *T* = 0.07 are retained, whereas the one at *T* = 0.06 is discarded.

Extracellular recordings of single-neuron activity are done by placing electrodes in brain tissue. The electrical potential changes measured at the electrode tip reflect the spiking activity of neurons close enough to the electrode plus background activity elicited by neurons further away from the tip (black trace in Fig. 1*A*,* top*). In principle, the spikes fired by a neuron recorded by a given electrode have a distinct shape. This is mainly determined by the morphology of the dendritic tree of the neuron, the distance and orientation relative to the recording site, the distribution of ion channels, and the properties of the extracellular medium (Gold et al. 2006). Spike-sorting algorithms detect these spikes (Fig. 1*A*,* top*) and, using features extracted from the waveforms, group them into clusters corresponding to the putative activity of different neurons (Fig. 1*A*,* bottom*,* right*) (Lewicki 1998; Quian Quiroga 2007). The importance of spike sorting is stressed by the fact that nearby neurons recorded from the same electrode can respond to completely different things, and, therefore, it is crucial to know which spike corresponds to which neuron. This is the case, for example, in the human and the rat hippocampus, where nearby neurons fire to unrelated concepts in the first case (De Falco et al. 2016; Rey et al. 2015a) and to distant place fields in the latter (Redish et al. 2001).

The simultaneous recording of a large number of electrodes, thus accessing the activity of populations of neurons, is becoming an essential tool for understanding complex behaviors and network properties in the brain (Quian Quiroga and Panzeri 2009). Silicon probes have been developed in the last 10 years (Blanche et al. 2005; Buzsáki 2004; Csicsvari et al. 2003), and large multielectrode arrays with up to thousands of electrodes are already being used for recording in retinal patches (Litke et al. 2004), cell cultures (Lambacher et al. 2011), or brain slices (Frey et al. 2009). Furthermore, a large number of channels is presently used to record from local circuits in behaving animals (Berényi et al. 2014). With such high channel count, the manual supervision of each single channel might turn into a very time-consuming task. In addition, it is well known that the subjectivity introduced by the human intervention creates an additional source of sorting errors (Harris et al. 2000; Wood et al. 2004). In parallel to these advances, there are also several cases in which single-electrode recordings are still routinely used, with automatic implementations being critical to avoid potential subjective biases of manual solutions. This is, for example, the case in invasive human recordings performed with depth electrodes implanted in patients with epilepsy (Rey et al. 2015a) or chronic implants for brain-machine interface (Homer et al. 2013). In this context, the unsupervised classification of single units, both from single-channel recordings and large-electrode arrays, has become the bottleneck to fully reach the potential of extracellular recordings (Harris et al. 2016; Rey et al. 2015b).

Many spike-sorting algorithms have been developed in the past years (see Rey et al. 2015b for a review). Some of these methods are based on Bayesian statistical frameworks, relying in some cases on a Gaussian model of the distribution of the spike waveforms (Harris et al. 2000; Rossant et al. 2016). Past studies have shown the non-Gaussian variability of the spike shapes and the nonstationarity of the extracellular recordings, attributable to, for example, small electrode drifts (Fee et al. 1996; Harris et al. 2016) or the presence of bursting cells (Henze et al. 2000), and motivated the development of nonparametric approaches. One such method is *Wave_clus* (Quian Quiroga et al. 2004), a spike-sorting algorithm that uses wavelet decomposition to extract features of the spike waveforms and superparamagnetic clustering (SPC) to cluster the spikes in this feature space. However, *Wave_clus* and the other most widely used algorithms are semiautomatic in practice, requiring manual tuning of a first automatic solution to achieve good performance. To tackle this problem, we analyzed the actions typically performed by *Wave_clus* users to optimize spike-sorting performance. We then implemented a set of heuristic modifications to the algorithm to reproduce the users’ actions in a fully automatic way. Moreover, we present an automatic criterion for selecting the number of features, i.e., of wavelet coefficients, to be used for clustering, given that, intuitively, only a few features are required to separate relatively few neurons, and more features are necessary for recordings containing a larger (and in principle unknown) number of neurons.

We evaluated the performance of the proposed method using both real and simulated single-channel and tetrode recordings. With the simulated data from single channels, the proposed algorithm significantly outperformed other automatic sorting algorithms, as well as the experts’ manual solution with the former *Wave_clus* implementation. With the simulated tetrode recordings, the new algorithm also outperformed other recently introduced methods, as quantified by the number of hits (i.e., correctly identified clusters) and false positives. With the real single-channel recordings, the algorithm retrieved, in a fully unsupervised way, ~95% of the clusters isolated by the sorting experts while keeping a low number of false positives. When assessed with real tetrode recordings, the new automatic algorithm outperformed the experts’ manual solution with the former *Wave_clus* implementation.

## MATERIALS AND METHODS

### Former Wave_clus Implementation

Our new method is based on the former MATLAB implementation of *Wave_clus*, introduced in Quian Quiroga et al. (2004). As with other spike-sorting methods, this algorithm has four main steps, including filtering, detection, feature extraction, and clustering (Rey et al. 2015b). Zero-phase filtering is done by using a second-order bandpass elliptic filter in the range of 300–3,000 Hz. Spike detection is performed by setting a threshold as

(1) $$\text{Thr}=5{\text{σ}}_{n},\;with\;{\text{σ}}_{n}=\text{median}\left\{\frac{\left|x\right|}{0.6745}\right\}$$

where *x* is the bandpass filtered signal (Rey et al. 2015b). An example of a signal filtered for detection and thresholding is shown in Fig. 1*A*,* top*. For each detected spike, 64 samples are saved for further analysis, aligned to their maximum at data point 20. To avoid spike misalignments attributable to low sampling, spike maxima location is refined by using cubic spline-interpolated waveforms with 320 samples. After realignment, the waveforms are downsampled back to 64 points.

Feature extraction is done using a four-scale multiresolution decomposition with a Haar wavelet, resulting in 64 wavelet coefficients associated with each detected spike. To assess the ability of each coefficient to separate different clusters, the algorithm uses a Lilliefors test (a normality test based on the Kolmogorov-Smirnov test), retaining the 10 most significant ones. To minimize the effect of outliers in the test, only values within three standard deviations are considered for each coefficient. For tetrode recordings, the spikes detected at each channel were concatenated. Then, if *Nch* channels are used with 64 samples per spike, there will be a total of Nch*64 wavelet coefficients, from which *Wave_clus* picks the Nch*10 most significant ones.

Finally, a nonparametric clustering is performed in the feature space using SPC. SPC is an unsupervised approach in which the grouping of points into clusters depends on nearest-neighbor interactions (Blatt et al. 1996, 1997). SPC generates a family of solutions as a function of a parameter called the temperature, which is the key parameter to determine how clusters are split (Domany et al. 1999). In analogy with models in statistical mechanics, at low temperatures, all data points are highly correlated and are therefore grouped into a single or relatively few clusters. On the other hand, at high temperatures, the correlations are too weak, and clusters break up into many groups with very few members in each group. At a certain temperature range between these two extremes, natural clusters appear (i.e., the superparamagnetic regime), and only points corresponding to data from relatively high-density regions are grouped. With SPC, for each temperature a different data partition is generated, resulting in a “temperature plot” (Fig. 1*A*,* bottom*,* left*). *Wave_clus* uses a range of temperatures from 0 to 0.25 in increments of 0.01, where each temperature is labeled as *T_i_*, with *i* = {0, …, 25}. At each temperature, clusters are sorted in decreasing order with respect to their size; thus, at temperature *T_i_* the largest cluster is denoted as $C_{1}^{T_{i}}$, the second largest as $C_{2}^{T_{i}}$, etc.

In *Wave_clus*, clusters are automatically identified according to a thresholding procedure based on their size. The idea is that, as the temperature is increased, new clusters appear. *Wave_clus* selects the highest temperature where at least one of the sorted clusters $C_{i}^{T_{n}}$ increases its size a minimum number of spikes (parameter *N_inc*) (Quian Quiroga et al. 2004). At this temperature, the cluster that increased its size by at least *N_inc* spikes and all the clusters with a larger size are selected. The parameter *N_inc* is introduced to avoid overclustering, i.e., choosing a very high temperature at which data is grouped into many clusters with a few members each. Once the clusters have been identified, a template-matching procedure is used to assign the remaining unclassified waveforms. For each cluster, the centroid (mean waveform) and a measure of its total variance, σ*_T_*, are computed, where ${\text{σ}}_{T}=\sqrt{\overset{64}{\underset{i=1}{\sum }}var\left(x_{i}\right)}$, with $var\left(x_{i}\right)$ denoting the variance at the *i*-th sample across the waveforms of a given cluster. Each spike is then assigned to the cluster with the smallest Euclidian distance to its centroid, as long as this distance is smaller than 3σ*_T_* (waveforms with a larger distance to the centroid are considered to be noise).

### New Wave_clus Implementation

To improve the former implementation of *Wave_clus*, we propose the use of heuristic modifications that are inspired by the actions that are usually taken by experts to optimize the automatic solution given by the previous implementation. The main limitation of the previous automatic implementation is that a single temperature is chosen for clustering, and, in many cases, there are clusters appearing at different temperatures. The rationale of the new implementation is to choose all putative clusters from the different temperatures in the temperature plot (peak selection step) and then get rid of false positives by avoiding double detections (inclusion criterion) (as the same clusters may appear at different temperatures) and also avoiding overclustering at large temperatures (regime border detection). A final improvement is the introduction of an automatic criterion to select a variable number of features (i.e., wavelet coefficients) for clustering.

Finally, we also included some improvements in the implementation of the algorithm to reduce the computing time. Specifically, we used a MEX implementation of the MATLAB function *filtfilt* for the detection process and eliminated time-consuming “for loops” in the interpolation procedure for spike detection and the wavelet decomposition of the detected waveforms (using optimized matrix manipulations). The codes and documentation of the new sorting implementation are available on GitHub (https://github.com/csn-le/wave_clus).

#### Peak selection in the temperature plot.

We first select every cluster $C_{i}^{T_{n}}$, where its size $\left|C_{i}^{T_{n}}\right|$ is increased by at least *N_inc* spikes as the temperature is increased from *T*_*n*−1_ to *T_n_*, i.e.,

(2) $$\left|C_{i}^{T_{n}}\right|-\left|C_{i}^{T_{n-1}}\right|\geq N\_inc,\;\text{with}\;\left|C_{i}^{T_{-1}}\right|=0$$

To consider other relevant members of a partition at temperature *T_n_* in which cluster $C_{i}^{T_{n}}$ has been selected by the peak criterion, all clusters with a larger size (i.e., all clusters $C_{j}^{T_{n}}$ with *j < i*) are also selected.

#### Regime border detection.

The end of the superparamagnetic regime is normally associated with an abrupt decrease of the principal cluster as we transition from $C_{1}^{T_{B-1}}$ to $C_{1}^{T_{B}}$, with the appearance of several small clusters (overclustering). However, in some cases, before reaching the end of the regime, there is an abrupt decrease in the principal cluster in conjunction with the appearance of a large new and relevant cluster, typically being the second largest (*C_2_*). For this reason, we first define the largest increment at a certain temperature as $LI_{T_{i}}=\text{max}\left(\left|C_{j}^{T_{i}}\right|-\left|C_{j}^{T_{i-1}}\right|\right)$, for *j* > 1. We then find the temperature *T_B_* as the minimum *T_i_* fulfilling the condition

(3) $$T_{B}=\min\left(T_{i\;}\right)\text{for which}\;\frac{\left|C_{1}^{T_{i}}\right|+LI_{T_{i}}}{\left|C_{1}^{T_{i-1}}\right|}<Thr\_border$$

with *Thr_border* being a threshold parameter (we used a value of 0.4 in our implementation, although we have seen that the results are not changed for 0.25 < *Thr_border* < 0.45). This way, when the ratio is small, the principal cluster is largely split beyond what could have been related with a new cluster (associated with the increment *LI*).

#### Inclusion criterion.

We first define the overlapping coefficient

(4) $$O_{i,j}^{T_{n},T_{m}}=\frac{\left|C_{i}^{T_{n}}\cap C_{j}^{T_{m}}\right|}{min\left(\left|C_{i}^{T_{n}}\left|,\right|C_{j}^{T_{m}}\right|\right)}$$

This coefficient ranges between 0, if the clusters are completely different (i.e., they do not share any spike), and 1, when they are the same, or one of them is a subset of the other. To avoid considering the same cluster of spikes twice (from different temperature partitions), if the value of $O_{i,j}^{T_{n},T_{m}}$ is not lower than a constant *k_O_*, only the cluster at the higher temperature is kept. In our implementation, we chose *k_O_* = 0.9. We also observed that the performance of the algorithm is not affected by this parameter choice in a wide range of values (we verified this for *k_O_* between 0.50 and 0.95).

#### Feature selection criterion.

**Fig. 2.**
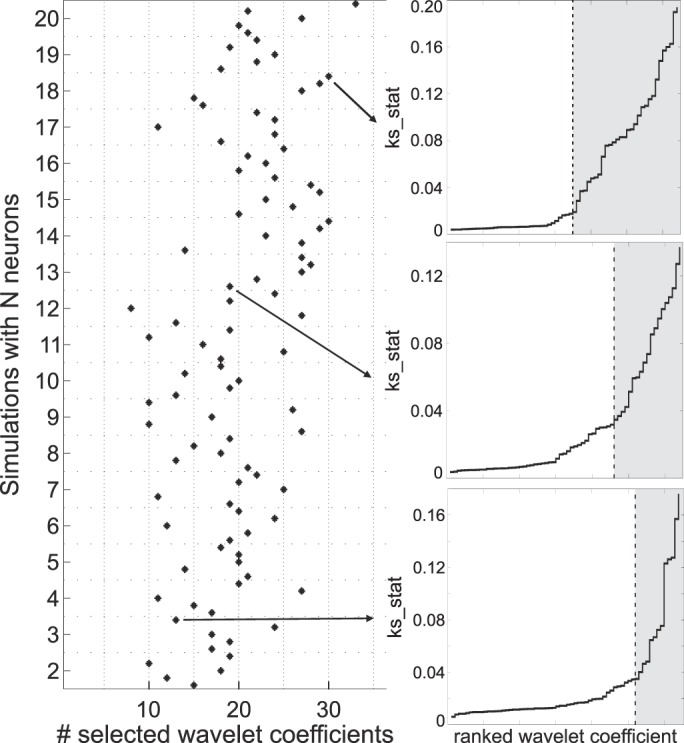
New criterion for feature extraction applied on the data set *Sim2*. *Left*: number of selected wavelet coefficients is shown for each simulation, with the simulations being sorted according to the number of simulated single units. There is a clear correlation between the number of chosen coefficients and the number of simulated units (Spearman correlation, ρ = 0.42, *p* ~10^−5^). *Right*: 3 particular examples are shown, depicting the sequence of *ks_stat*, i.e., the statistic associated with the Lilliefors test. The shaded gray area covers the coefficients that were selected on each particular example. Notice the change in the slope of the curve at the point marked by the vertical dashed line.

In the new implementation, we introduced an automatic data-driven selection of the relevant features to be used for clustering. For each wavelet coefficient, the Lilliefors test returns a test statistic *ks_stat*. The coefficients with large values of *ks_stat* tend to be associated with multimodal distributions and represent the ones that should be selected for clustering. First, we sort the set of *ks_stat* values in ascending order, leading to the sequence {*ks_stat_sorted*}. We empirically found that this sequence tends to increase exponentially. We introduce a smooth estimate of the first derivative of the sequence by computing a difference quotient through a sliding window with a span of 10 samples (i.e., the first quotient is (*ks_stat_sorted*(10)–*ks_stat_sorted*(1)) / 10), normalizing by the number of coefficients and the maximum value of *ks_stat*. Then, we look for the first point where the estimate is >1 for three consecutive samples. This is an estimate of the point colloquially referred to as “the knee of the exponential,” which is indeed where the radius of curvature reaches its minimum. Finally, all the coefficients to the right of the estimated knee are selected, i.e., those where *ks_stat > ks_stat_sorted*(*knee*). Examples of the criterion can be seen in Fig. 2 and Fig. 7*A*.

We have implemented the automatic feature selection criterion in all cases, except for the real single-channel recordings from the human hippocampus (see below), given that, in this case, we wanted to compare the automatic sorting with the actions taken by the expert sorters (who did the sorting with a fixed number of wavelet coefficients).

### Real Data Sets

#### Single channel recordings in the human medial temporal lobe.

We used recordings in the human medial temporal lobe of five patients implanted with depth electrodes for epilepsy diagnosis (Quian Quiroga et al. 2008). Each electrode probe had a total of nine microwires at its end, eight active recording channels, and one reference. The differential signal from the microwires was amplified by a 64-channel Neuralynx system, filtered between 1 and 9,000 Hz and sampled at 28 kHz.

This data set was sorted with the former *Wave_clus* implementation (i.e., with a fixed number of 10 wavelet coefficients), which was then manually optimized by an expert. It should be highlighted that we do not have ground truth with such real data. However, the first goal of the new method is to automatically replicate the steps manually done by the expert to optimize the sorting outcomes. Therefore, the expert’s solution was taken as the desired solution to assess the performance of the novel implementation introduced in this work. In other words, the goal with this data set was to fully automatize the manual optimization process to bypass the need of the user’s intervention. A total of 200 recordings from the human medial temporal lobe were used (each ~15 min long), where at least two clusters were identified.

Using the same temperature plot for the expert sorters and the automatic implementation, we could quantify whether the latter selected the same clusters (peaks in the temperature plot) as the expert sorters. The true positive rate (TPR) was computed as the ratio between the number of clusters identified by the algorithm that corresponded to a cluster selected by the expert and the total number of clusters identified by the expert. In addition, the false positive rate (FPR) was calculated by counting the number of clusters identified by the algorithm in the temperature plot that did not correspond to a cluster selected by the expert, divided by the maximum number of false positives generated over all recordings, algorithms and *N_inc* values. This way, for each algorithm and *N_inc* value (ranging from 10 to 60 in steps of 5), we computed the TPR and FPR of the real data set, allowing us to construct receiver operating characteristic (ROC) curves. In addition, we quantified for each ROC curve the area under the curve (AUC) as a measure of performance of each algorithm.

#### Tetrodes.

We also evaluated the performance of the algorithm with tetrode recordings. A tetrode recording from a locust was kindly provided by Ofer Mazor and Gilles Laurent (Perez-Orive et al. 2002). Moreover, we used a set of 20 recordings with tetrodes implanted in 4 patients with intractable epilepsy, using the same procedures as reported in Quian Quiroga et al. (2008). Although we do not have ground truth with real data, the solutions from the different algorithms were analyzed by an expert, who assessed whether clusters corresponded to different single units based on spike shape differences and standard criteria, such as cross-correlation of spike times and the presence of refractory period violations.

### Simulated Data Sets

#### Single-channel simulations.

Two different publicly available simulated data sets were used to evaluate the performance of the proposed method against ground truth. The first one (*Sim1*) was introduced in Quian Quiroga et al. (2004) (available at http://www.vis.caltech.edu/~rodri/Wave_clus/Simulator.zip), and it comprises four simulations, each with three different neurons, under different levels of background noise. The second set (*Sim2*) was presented in Pedreira et al. (2012) (available at https://www135.lamp.le.ac.uk/hgr3/), and it includes several simulations done with a varying number of neurons, ranging from 2 to 20. The rationale for using up to 20 neurons was to test the performance of the algorithm in challenging scenarios, especially considering the fact that current spike-sorting algorithms tend to detect fewer neurons than they should, based on anatomical and physiological considerations (Henze et al. 2000; Pedreira et al. 2012). In addition, three independent experts performed manual supervision on this set optimizing the results obtained with the former implementation of *Wave_clus*. We averaged their performance to get a single “Expert” score per simulation.

All the simulated waveforms were clustered using the two different *Wave_clus* implementations, and the number of hits and false positives were quantified as in Pedreira et al. (2012) and Niediek et al. (2016). A selected cluster was considered as a hit when >50% of its spikes were correctly identified. Choosing other definitions (i.e., considering >70 or 80% of the spikes) gave qualitatively similar results. Selected clusters that were not a hit were labeled as false positives. Missed clusters were calculated as the number of simulated units minus the number of hits. The multiunit clusters were not considered in the hit calculations but were considered false positives if <50% of their spikes came from the multiunit.

With single-channel recordings, we compared the performance of our algorithm to the one given by other recently proposed methods. For performance comparison with other algorithms, we used Klusta (Rossant et al. 2016; version February 2017) and Combinato (Niediek et al. 2016; version April 2018). In particular, Klusta is an improved implementation of the former Klustakwik (Harris et al. 2000), a spike-sorting algorithm that extracts waveform features through principal component analysis and uses a Gaussian mixture model to perform the clustering of the data. Some of its detection parameters were modified from their default values to achieve a detection performance comparable to the one with *Wave_clus*. Particularly, we used filter_low = 300, filter_high = 3,000, threshold_strong_std_factor = 4, threshold_weak_std_factor = 3.8, extract_s_before = 24, and extract_s_after = 40. Its default number of principal components used for feature extraction is 3. However, we observed in our simulations that a larger number of hits was achieved with 10 (*p* ~10^−3^), without differences in terms of the number of false positives, so the results we show for Klusta are based on 10 principal components. Combinato is a recently introduced algorithm that also uses SPC for clustering the data but includes an iterative reclustering of large clusters. As the authors tested Combinato with the same simulated data set we used here, we chose the set of parameters reported in their paper.

#### Tetrodes.

We created a set of simulated tetrode recordings using Neurocube (Camuñas-Mesa and Quian Quiroga 2013), a hybrid modeling approach that uses a detailed compartmental model to simulate the contribution of neurons near the recording electrode, and previously recorded spike shapes to generate the background noise (with their amplitude scaled inversely to the squared distance between the neuron and the recording site). To simulate multiunit activity (with detailed neuron models; 1:4 ratio between interneurons and pyramidal neurons), we used 8% of active neurons located between 60 and 150 μm away from the tetrode (using a density of 300,000 neurons/mm^3^), with their spike time intervals drawn from an exponential distribution (mean = 3 Hz). A certain number of pyramidal single units (ranging from 11 to 20) were randomly placed between 10 and 40 μm away from the tetrode with their spike time intervals drawn from an exponential distribution (mean = 3 Hz). The diameter of the electrodes comprising the tetrode was of 20 μm, with 40-μm spacing. Each simulation was 15 min long. Waveforms generated by multiunit neurons that had a peak larger than five times the standard deviation of the noise and were <1 ms from the spike of a single unit were subtracted from the signal to reduce the level of closely overlapping spikes. Following spike detection, the spikes associated with the multiunit activity represented between 50% and 70% of the total number of detected spikes, which is consistent with what is typically observed in real data.

Performance was assessed with the same criteria of hits and false positives used with the single-channel data set. We compared our results with other recently proposed algorithms that were specifically developed to sort multichannel recordings, MountainSort (Chung et al. 2017; version 0.11.6), Kilosort (Pachitariu et al. 2016; version February 2018), and SpyKING CIRCUS (Yger et al. 2018; version 0.6.4). We also included Klusta, for which we used the same parameters as in the single-channel simulated set. For the other algorithms, we used their default parameters (in SpyKING CIRCUS, we enabled “auto-mode merging” as suggested for a fully automatic implementation and did not use the spatial whitening option, as it led to a large decrease in detection performance).

## RESULTS

### Description of the New Sorting Implementation

The key advantage of the new *Wave_clus* implementation is that it automatically selects clusters from different temperatures. This allows identifying units with different firing rates and spike shape characteristics that differ in their density and location within the feature space. To illustrate this point, Fig. 1*A* shows a segment of real data (high-pass filtered), in which the spikes associated with different units can be observed. On the basis of the different data partitions generated by the SPC algorithm, which are shown in the temperature plot in Fig. 1*A*,* bottom*,* left*, an expert user selected four clusters (3 single units and 1 multiunit) at different temperatures. To achieve this automatically, a three-step procedure was used (see materials and methods for details). First, putative clusters were selected at different temperatures. It should be noted that a similar idea of finding peaks at different temperatures in the temperature plot has also been introduced in Niediek et al. (2016). Second, peaks from very high temperatures were eliminated to avoid overclustering. Figure 1*B* shows an example of the identification of *T_B_*, with the gray shaded area indicating all the temperatures that are disregarded and which may have contributed with spurious peaks. Third, the algorithm quantified the overlap between pairs of clusters to avoid double counting when considering the same spikes at different temperatures. Figure 1*C* shows an example with three peaks, which in this case were obtained because the larger cluster at *T* = 0.06 was split in two at *T* = 0.07. The overlapping coefficient between the clusters at both temperatures was equal to 1 in both cases, thus correctly showing that these three peaks correspond to the same cluster split in two; therefore, the clusters at *T* = 0.07 were retained, and the one at *T* = 0.06 was discarded.

A final improvement compared with the former implementation is that the new algorithm automatically selects the number of relevant features (i.e., wavelet coefficients) to be used for clustering. Previously, we selected a fixed number of coefficients, 10 out of a total of 64 wavelet coefficients, which are the ones differing the most from a Gaussian distribution, according to the Lilliefors test. Figure 2 shows the number of coefficients selected with the proposed method on each simulation of data set *Sim2* (see materials and methods). This data set comprises five simulations with *N* single units, with *N* ranging from 2 to 20. We observe that the number of selected coefficients increases with the number of neurons in the simulation (Spearman correlation, ρ = 0.42, *p* ~10^−5^). In other words, with few neurons, relatively few coefficients will be enough to separate the associated clusters, but, as more neurons are present in a recording, it is necessary to consider more coefficients. However, if we use all 64 coefficients, we will also consider those that capture just noise, thus decreasing clustering performance (see below). The proposed criterion automatically selects the coefficients in a data-driven way. Three particular examples can be seen in the inset of the figure, showing the dynamics of the statistic of the Lilliefors test and the estimated knee of the exponential (see materials and methods), from which all coefficients to the right were selected.

### Performance with Real Single-Channel Recordings

**Fig. 3.**
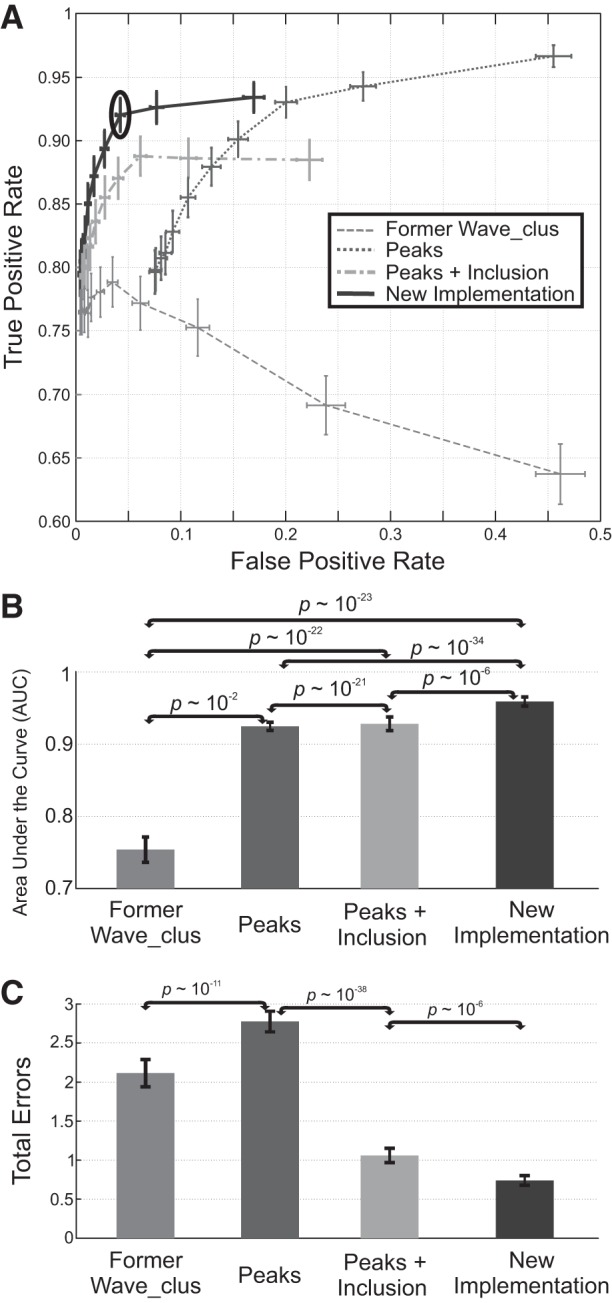
Performance of the proposed improvements with a real data set. *A*: for each algorithm implementation, the parameter *N_inc* took values from 10 to 60 in steps of 5 (as *N_inc* is increased, we moved on the figure from right to left). In each case, the true and false positive rates were computed, allowing us to plot mean and SE across the 200 recordings analyzed. For the inclusion criterion, we used *k_O_* = 0.9, and the border of the superparamagnetic regime was computed using *Thr_border* = 0.4. The new *Wave_clus* implementation (solid line) includes the peak selection, inclusion, and regime border detection criteria. The black oval marks its performance with *N_inc* = 20, which is the final value chosen for the new implementation. *B*: mean and standard error of the mean of the area under the curve (AUC) across the 200 recordings. Paired sign tests were used to evaluate the statistical difference across implementations. *C*: mean and standard error of the mean of the total number of errors (i.e., misses + false positives) across the 200 recordings. Paired sign tests were used to evaluate the statistical difference across implementations.

We first evaluated the performance of the new method with real data collected from recordings in the human medial temporal lobe (see materials and methods). The data set comprises 200 recordings with at least two units identified by an expert user. We used *N_inc* values from 10 to 60 in steps of 5. This way, as shown in Fig. 3*A*, we could compute an ROC curve for each sorting implementation we tested (see materials and methods).

With the former *Wave_clus* implementation (dashed line), increasing *N_inc* led to an increase of the TPR, with a reduction of the FPR, until *N_inc* = 25; then the TPR stabilized at 75–80%. When the proposed peak selection method was included (dashed line), low values of *N_inc* were associated with high TPRs but also with high FPRs. Increasing *N_inc* reduced the number of false positives (at the expense of also reducing the TPR) although the FPR remained relatively large compared with the former *Wave_clus* implementation. The addition of the inclusion criterion led to the ROC being shifted to the left (dashed line) but with a reduction in the TPR (although still with TPRs 10% higher than with the former *Wave_clus* implementation) attributable to overclustering. Finally, the addition of the regime border detection reduced the chance of overclustering (solid line), boosting the TPR an extra 5% while maintaining low FPRs. Figure 3*B* shows the statistics on the AUC associated with the different sorting implementations. An AUC closer to 1 is related to a better performance. The addition of each of the proposed improvements leads to a significant increase in the AUC and the new implementation, including the peak selection, inclusion, and regime border detection criteria gave the best performance. In Fig. 3*C*, we further evaluated the contribution of each step in terms of the number of total errors, i.e., misses + false positives. From the former *Wave_clus* implementation, the addition of the peak selection alone actually led to a significant worsening of the performance attributable to the high number of false positives generated (notice how the ROC in Fig. 3*A* is shifted to the right). However, when the inclusion criterion was also added, the number of errors dropped significantly, outperforming both peaks and the former *Wave_clus* implementation. Finally, the further addition of the regime border detection led to a further improvement in performance, with the resulting new implementation exhibiting a significantly lower number of errors than any of the other alternatives.

This data set allowed us to evaluate the performance of the improvements associated with the actions taken by the experts and to assess the contribution of each of them. As a result, we found that, by choosing *N_inc* = 20 with the new implementation, we automatically detected almost 95% of the clusters manually detected by the expert sorters with an average of less than one false positive. This represents a 15% increase in TPR with a similar low FPR compared against the former *Wave_clus* implementation.

### Performance with Simulated Single-Channel Recordings

#### Results with data set Sim1.

The advantage of using simulated data is that it provides ground truth to evaluate the performance of a spike-sorting implementation. First we used the data set introduced in Quian Quiroga et al. (2004), in which three neurons were simulated in each recording with different waveforms (in the simulations named “Easy” waveforms were very different from each other, whereas in the ones labeled “Difficult” they were very similar) and with different levels of background noise. For each simulation, we evaluated the number of hits (see materials and methods) with the former and new *Wave_clus* implementations using a variable number of wavelet coefficients (with the new implementation using the peak selection, inclusion, and regime border detection criteria introduced in *Description of the New Sorting Implementation*). Table 1 shows that, as shown in Quian Quiroga et al. (2004), the former *Wave_clus* implementation using 10 wavelet coefficients was able to cluster all three simulated units in all but one case (the hardest one in terms of noise level and waveform similarity). However, when no dimensionality reduction was performed, i.e., when all 64 wavelet coefficients were used, the performance dropped significantly (former 10 wavelet coefficients vs. 64 wavelet coefficients, paired sign test, *P* = 0.008). This drop is particularly evident for the cases with large noise levels, as several coefficients captured noise features, compromising the performance of the clustering algorithm. The new implementation exhibited a similar performance with 10 wavelet coefficients and also showed an analogous drop in performance when all 64 wavelet coefficients were used (new 10 wavelet coefficients vs. 64 wavelet coefficients, paired sign test, *P* = 0.016). Finally, when the selection of a variable number of wavelet coefficients was used, the performance was the same as with 10 wavelet coefficients. As discussed below, this is due to the fact that, for a relatively low number of neurons, 10 wavelet coefficients already resulted in nearly optimal performance.

**Table 1.** Performance of the proposed implementation with the simulated data set *Sim1*

	Former *Wave_clus*		New Implementation		
	No. of coefficients		No. of coefficients		
Simulation	10	64	10	64	variable
Easy1_noise005	3	3	3	3	3
Easy1_noise010	3	3	3	3	3
Easy1_noise015	3	3	3	3	3
Easy1_noise020	3	3	3	3	3
Easy1_noise025	3	3	3	3	3
Easy1_noise030	3	3	3	3	3
Easy1_noise035	3	2	3	3	3
Easy1_noise040	3	1	3	1	3
Easy2_noise005	3	3	3	3	3
Easy2_noise010	3	3	3	3	3
Easy2_noise015	3	3	3	3	3
Easy2_noise020	3	1	3	1	3
Diffi1_noise005	3	3	3	3	3
Diffi1_noise010	3	3	3	3	3
Diffi1_noise015	3	2	3	0	3
Diffi1_noise020	3	0	3	0	3
Diffi2_noise005	3	3	3	3	3
Diffi2_noise010	3	2	3	2	3
Diffi2_noise015	3	2	3	1	3
Diffi2_noise020	2	0	2	0	2

Each row denotes a simulation of 3 different neurons with different levels of background noise. When the waveforms were different enough, they are labeled as “Easy,” whereas, when they were similar, they were labeled as “Difficult.” The peak amplitude of each spike was normalized to 1, and the standard deviation of the background noise was varied from 0.05 to 0.20 (or 0.40 for “Easy1”). The table shows the number of neurons (up to 3) correctly detected in each case. In the former *Wave_clus* implementation, the performance with 64 wavelet coefficients (WC) was significantly worse than with 10 WC (paired sign test, *P* = 0.008). The same behavior was also seen with the new implementation with a fixed number of WC (10 WC vs. 64 WC, paired sign test, *P* = 0.016), but no difference was seen between the new implementation with 10 or variable number of WC.

**Fig. 4.**
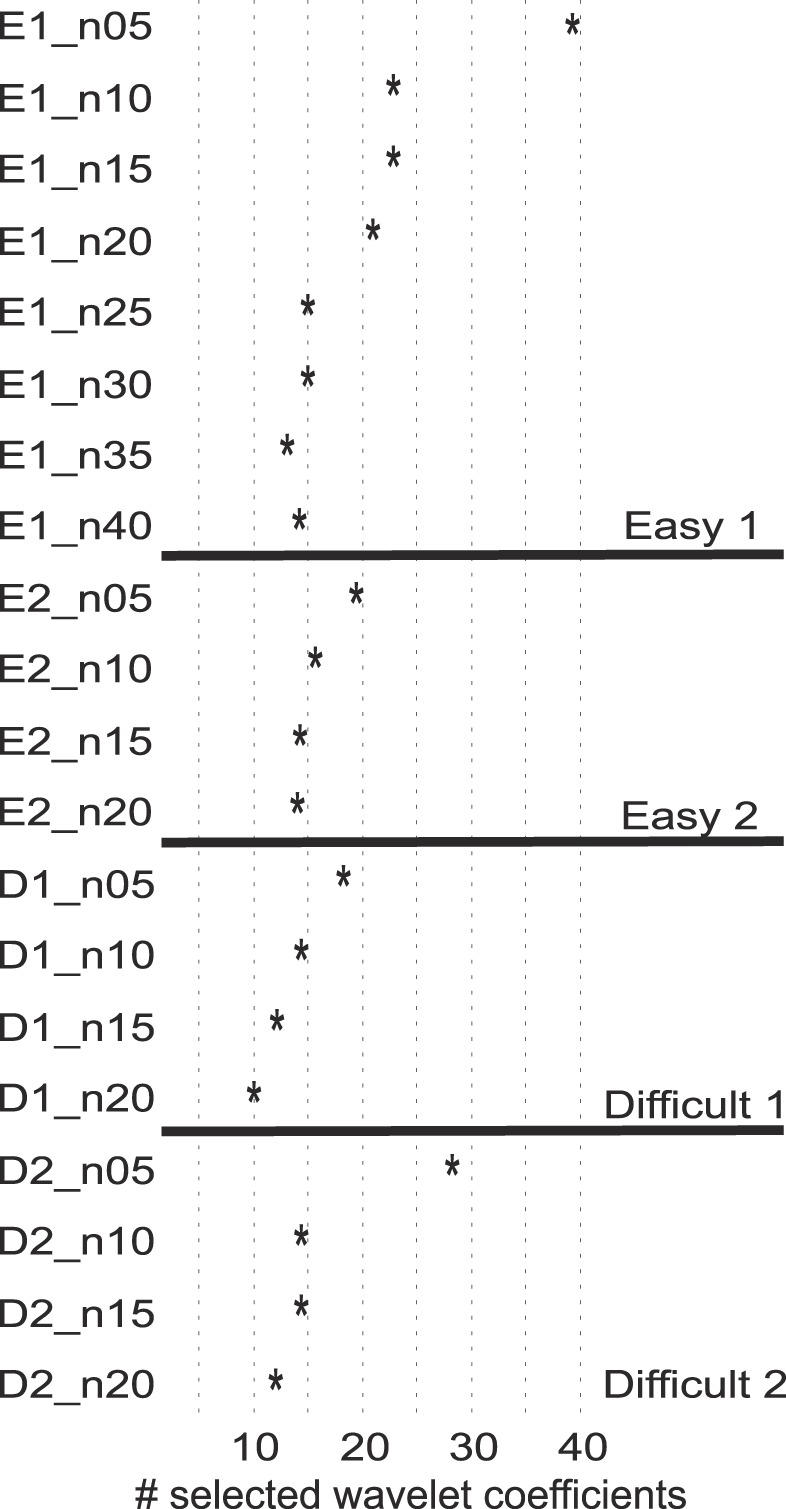
Selected wavelet coefficients for each simulation in the simulated data set *Sim1*. Each row represents a simulation with a given level of noise (e.g., E1_n10 depicts the simulation from the set “Easy1” with a noise level of 10). The number of selected coefficients is reduced as the noise level is increased. Particularly, for the simulations Easy1, created with 8 different noise levels, there was a significant correlation (Spearman correlation, ρ = 0.96, *p* ~10^−4^).

Figure 4 shows the number of automatically selected coefficients for each simulation. First, the easy simulations have more coefficients selected than the difficult ones; i.e., as the waveforms are easier to separate, more coefficients give information that differentiates between the different waveforms. Note that, for each set of simulations with varying degrees of noise, more coefficients were selected for the cases with relatively low noise levels (i.e., simulations labeled n05). Particularly, for the simulations “Easy1” (performed with 8 different levels of noise), there was a strong correlation between noise level and the number of coefficients selected (Spearman correlation, ρ = 0.96, *p* ~10^−4^). The other simulations have only four levels of noise, which is not sufficient to properly estimate the correlation. This trend can be explained by the fact that, when the noise level is low, several coefficients capture differences between spike shapes, whereas, with larger noise levels, more coefficients capture noise features that do not contribute to the separation of clusters and are therefore (correctly) not selected. Importantly, the new implementation with a variable number of wavelet coefficients has no a priori upper bound on the number of coefficients selected. However, Table 1 and Fig. 4 show that the number chosen did not go too high for the high noise conditions, preventing the drop of performance observed when all coefficients were selected (64 wavelet coefficients).

### Results with Data Set Sim2

**Fig. 5.**
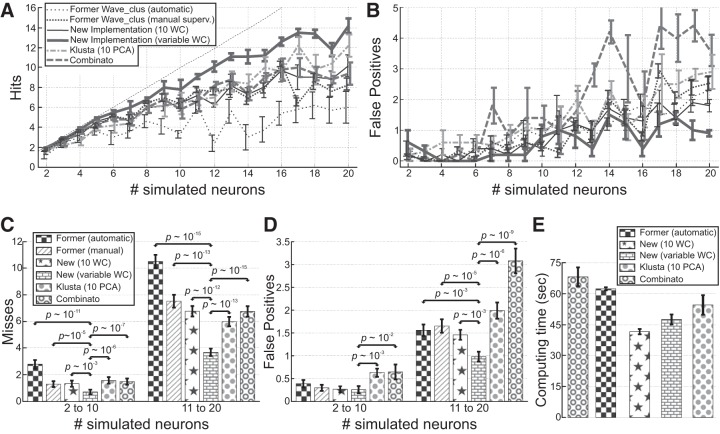
Performance of the proposed algorithm with the simulated data set *Sim2* (single channel). *A*: number of hits as a function of the number of simulated neurons for the different algorithm implementations. Means and SE across the 5 simulations with each number of simulated neurons are shown. The former *Wave_clus* was used in its automatic form and followed by manual supervision by an expert sorter. The curve for the new implementation with a fixed number of 10 wavelet coefficients (WC) is largely overlapping with the one from the experts. The new proposed method was also compared with Klusta, using 10 principal components (10 PCA), and Combinato, two recently introduced algorithms that are suitable for sorting single-channel recordings. *B*: same as in *A* but for false positives. *C*: means and SE of misses for each algorithm. The analysis was done for the subsets with a small (2 to 10) and large (11 to 20) number of neurons. Paired sign tests were used to evaluate the statistical difference across implementations. All the implementations showed better performance than the unsupervised former *Wave_clus*, whereas the proposed algorithm with a variable number of wavelet coefficients was significantly better than all other implementations. *D*: same as *C* but for false positives. Significant differences were found in the subset with a small number of neurons when compared with Klusta and Combinato, but, with more neurons, the proposed algorithm with a variable number of wavelet coefficients was significantly better than all other implementations. *E*: computing time of each algorithm using simulated single-channel recordings with 20,000 (±1%) spikes each. The new proposed algorithm has smaller computing times than Klusta and Combinato while achieving a much better performance, both in terms of hits and false positives.

Data set *Sim1* consisted of three simulated neurons of different spike shapes and with varying levels of noise. To test the performance of the algorithm in more challenging scenarios, we also used the data set *Sim2*, which consisted of 95 simulations, each with 2 to 20 neurons (see materials and methods). Three independent experts performed a manual optimization of the solution provided by the former implementation of *Wave_clus*. Figure 5, *A* and *C*, shows the performance of the algorithms in terms of hits and misses. As expected, the experts significantly improved the performance over the automatic solution from the former *Wave_clus* implementation, which suffered from the selection of a single temperature in the temperature plot. When we used a fixed number of wavelet coefficients (10), the unsupervised performance of the proposed method was not significantly different (both in terms of misses and false positives) to the one obtained by the experts (with the former *Wave_clus* implementation), thus showing that the ad hoc improvements introduced to mimic the expert’s actions were successful to deliver a fully unsupervised algorithm. It should be noticed that using only the peak selection step (without the inclusion and regime border detection) significantly deteriorated the performance of the algorithm because of a large number of false positives (as was the case with the real data presented in Fig. 3). Conversely, when the new implementation included the new feature selection criterion (i.e., using a variable number of wavelet coefficients), a further significant increase in the number of hits was achieved, outperforming the results obtained by the experts. In fact, the performance obtained by the experts saturates at ~8 hits, whereas the new algorithm managed to correctly isolate an average of 14 out of 20 simulated neurons from a single channel. Moreover, the use of variable coefficients led to significant improvements over the case with 10 wavelet coefficients, in terms of both misses (2 to 10, *p* ~10^−3^; 11 to 20, *p* ~10^−12^) and false positives (2 to 10, not significant; 11 to 20, *p* ~10^−3^).

### Comparison to Other Single-Channel Algorithms

Figure 5 also shows the comparison of performance with other recently introduced algorithms, Klusta and Combinato (see materials and methods). All the different implementations were statistically compared using paired sign tests. As the simulations contained more neurons, there was also more room for differentiating the performance between algorithms. For this reason, Fig. 5, *C* and *D*, compare the performance (using paired sign tests) separating the cases with a small (2 to 10) and large (11 to 20) number of neurons. When we compared the performance of Klusta and the experts, Klusta showed more hits in the set 11 to 20 (*p* ~10^−4^). In turn, Combinato showed no significant differences in the number of hits compared with the experts (although *P* = 5.4 × 10^−2^ in the set 11 to 20). Klusta and Combinato showed similar performance, with Klusta being better in the set 11 to 20 (*P* = 2.3 × 10^−2^). The new implementation of *Wave_clus* with a variable number of wavelet coefficients gave a further significant increase in the number of hits, outperforming all other methods with both small and large number of neurons (Fig. 5*C*).

We next considered false positives. As shown in Fig. 5, *B* and *D*, Klusta and Combinato showed a significant increase in the number of false positives compared with all other algorithms, regardless of whether the number of simulated neurons was small or large. In the set 11 to 20, the new *Wave_clus* implementation achieved a significantly smaller number of false positives than all other algorithms, with reductions of >50% compared with Klusta and Combinato.

Finally, we analyzed the computational cost of the different algorithms by using recordings with 20,000 spikes (±1%). Combinato detected 13,500 spikes, whereas the other algorithms detected 15,000 (in all cases, nearly all the single-unit spikes were detected). Combinato showed larger computing times than the former *Wave_clus* implementation. This is mainly due to the fact that every cluster identified with >1,000 spikes was subjected to a second run of SPC. The new *Wave_clus* with 10 wavelet coefficients (i.e., including the steps to get a fully automatic implementation and the improvements used for spike interpolation and wavelet decomposition) led to a large drop in computing time (~20 s on average). When the new feature extraction was included, computing times were increased by 6 s on average but were still smaller than those from the former *Wave_clus* and Combinato. The difference with the new implementation with 10 wavelet coefficients is fully accounted for by the time taken by SPC to cluster the spikes in a higher dimensional space (in this set, an average of 20 wavelet coefficients was used in the new *Wave_clus*). However, the small additional computing time was accompanied by a reduction of misses and false positives by almost 50%. Compared with Klusta and Combinato, reductions in misses and false positives were even larger and with less computing time. It should be noticed that, if recordings with more spikes were used, the computing time will not increase much because 20,000 spikes can be used for clustering with SPC, and the remaining ones can be assigned afterward by template matching.

### Performance with Real Tetrode Recordings

#### Results with an example tetrode recording in the locust.

**Fig. 6.**
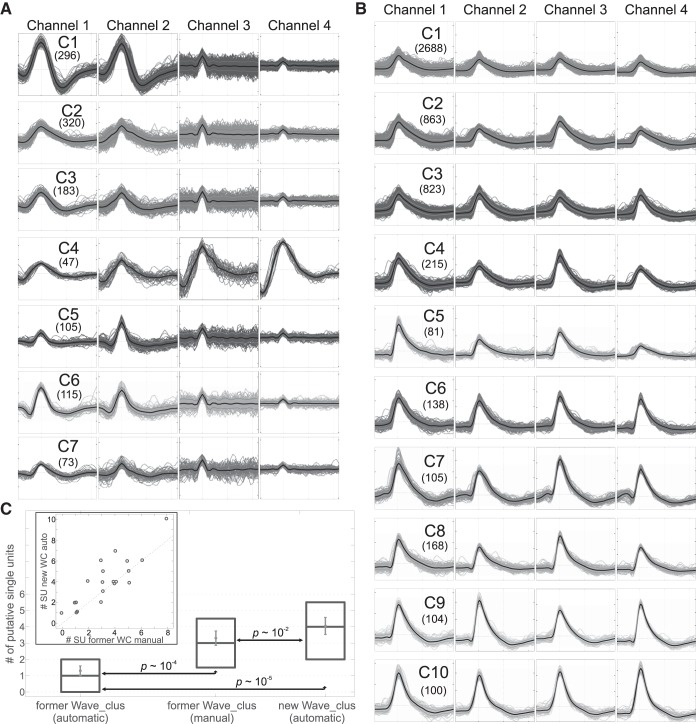
Performance of the proposed algorithm with real data from tetrodes. *A*: in a tetrode recording from a locust, the proposed algorithm was able to isolate 7 clusters (which were associated to putative single units according to an expert's opinion; see materials and methods for details). Spikes for each class on each recording channel are overlaid (total number of spikes per cluster shown in brackets), with the thick black line representing the mean waveform and the lighter ones associated with the SE. Horizontal dashed lines represent the voltage *y* = 0. Ticks on the *x*-axis are placed every 10 samples, whereas the separation in the *y*-axis is 1.0, 0.5, 0.2, and 1.0 for channels 1 to 4 (in arbitrary units). *B*: in a tetrode recording from the human hippocampus, the proposed algorithm was able to isolate 10 clusters. Ticks on the *x*-axis are placed every 20 samples, whereas the separation in the *y*-axis is 50 μV for all channels. *C*: analysis of a set of 20 tetrode recordings in the human hippocampus showed that the proposed algorithm was able to isolate significantly more putative units than the former implementation, even when followed by manual supervision by expert sorters. Means ± SE and boxplots (median ± 1st/3rd quartiles) are shown, as well as the results from paired sign tests. *Inset*: number of clusters isolated on each recording by the new automatic and former manual implementations.

We evaluated the performance of the proposed algorithm using real data recorded with tetrodes. Figure 6*A* shows the seven clusters isolated by the proposed algorithm from a tetrode recording in a locust, which displays clear differences in the spike shapes in the different channels. In contrast, the former automatic *Wave_clus* implementation only isolated two clusters, whereas following manual supervision it was possible to isolate six clusters (C5 and C7 remained merged in this case). Only 20 wavelet coefficients were automatically selected in the proposed implementation, in contrast to the 40 selected in the former implementation. Therefore, the 20 additional coefficients can be carrying information about noise, preventing the SPC from separating clusters C5 and C7.

#### Results with tetrode recordings in the human hippocampus.

We next analyzed a set of tetrode recordings in the human hippocampus. Figure 6*B* shows one example from that set, in which the new *Wave_clus* implementation was able to isolate 10 clusters. The former automatic *Wave_clus* led to two clusters of poorly isolated units (one with “small” spikes and another with “large” spikes); following manual supervision it was possible to increase the number of isolated clusters to seven. The new implementation automatically selected 57 wavelet coefficients, with the 17 additional coefficients allowing *Wave_clus* to isolate more clusters. Figure 6*C* presents the analysis over all 20 tetrode recordings. Although it is not possible to have ground truth with this data, we observe that the new implementation led to a larger number of identified putative single units (according to an expert's opinion), not only in comparison to the former automatic *Wave_clus* implementation, but also with respect to the former implementation optimized using manual supervision (but still using a fixed number of features). This latter difference can be attributed to the automatic selection of a variable number of wavelet coefficients.

#### Performance with simulated tetrode recordings.

**Fig. 7.**
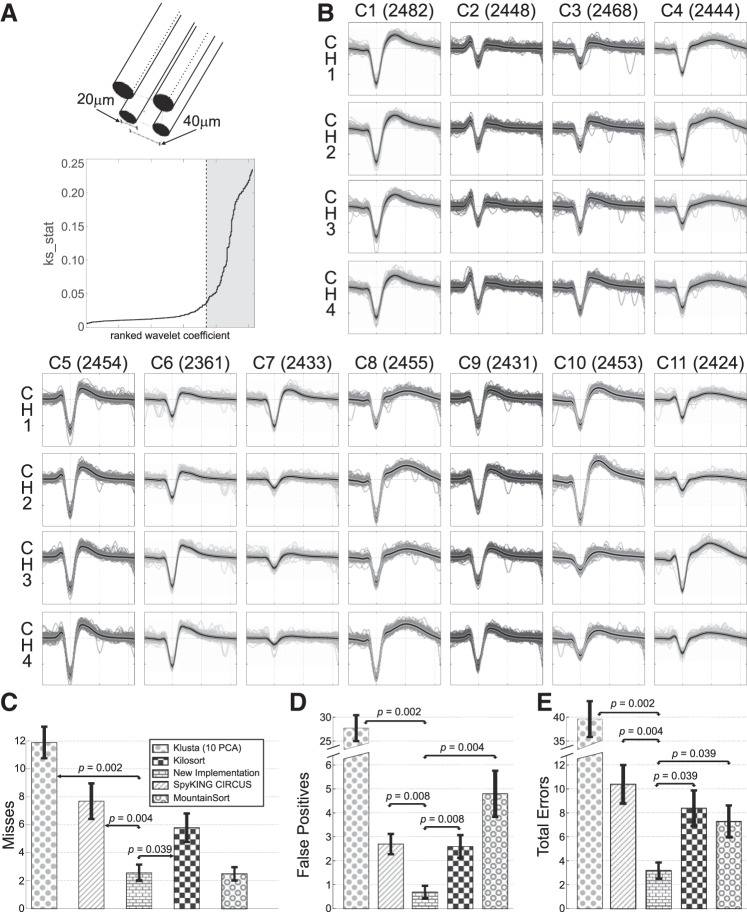
Performance of the proposed algorithm with the simulated tetrode data set. *A*: diagram of the simulated tetrode is shown on the top; each electrode had a 20-μm diameter, with a 40- μm separation between them. On the bottom, the coefficient selection for the simulation with 11 single units is shown (same conventions as in Fig. 2). In this case, a total of 72 coefficients were selected. *B*: results from the exemplar simulation with 11 single units are shown, as they were all properly isolated by the new implementation of *Wave_clus*. Spikes for each class on each recording channel are overlaid (total number of spikes per cluster shown in brackets), with the thick black line representing the mean waveform and the lighter ones associated with the SE. Ticks on the *x*-axis are placed every 20 samples, whereas the separation in the *y*-axis is 50 μV for all channels. *C*: mean and SE of misses for each algorithm. Paired sign tests were used to evaluate the statistical difference across algorithms. *D*: same as *C* but with false positives. The proposed algorithm with a variable number of wavelet coefficients was significantly better than all other implementations. *E*: total number of errors, i.e., misses + false positives, for the different algorithms. The new *Wave_clus* showed significantly fewer errors than all other algorithms, with an average of less than half the number of errors of the second best.

To quantify performance with tetrodes, we created 10 simulations including between 11 and 20 single units (see materials and methods). The top part of Fig. 7*A* shows a diagram of the simulated tetrode, in which each electrode had a 20-μm diameter with a separation of 40 μm between them. Figure 7*A*,* bottom*, shows the sequence of sorted values of the statistic *ks_stat* obtained from the Lilliefors test for the simulation with 11 single units. A total of 72 coefficients were automatically selected, which enabled the new *Wave_clus* to correctly isolate all 11 units without any false positives (the waveforms for the resulting clusters are shown in Fig. 7*B*).

#### Comparison to other algorithms.

To compare the performance of the different algorithms with the simulated data set, we analyzed the number of hits/misses and false positives, as previously done for the single-channel data set. All algorithms detected a similar number of spikes, capturing above 90% of the ones generated by the single units. Figure 7*C* shows that the proposed algorithm had a significantly lower number of misses compared with Klusta, SpyKING CIRCUS, and Kilosort (*P* = 0.002, *P* = 0.004, and *P* = 0.039, respectively) and a similar performance compared with MountainSort (the difference was not significant). However, as shown in Fig. 7*D*, differences with MountainSort and with all other algorithms were significant when considering the number of false positives. To quantify the overall performance, we computed the total number of errors. Figure 7*E* shows that the proposed algorithm had significantly fewer errors than all other algorithms (with at least half the number of errors compared with the algorithm with the second-best performance). Interestingly, there were no significant differences between MountainSort, Kilosort, and SpyKING CIRCUS (*P* > 0.34).

## DISCUSSION

We presented a fully automatic method that performed significantly better than a former *Wave_clus* implementation and also compared with other recently proposed unsupervised algorithms. Most of the modifications introduced were actually inspired by the actions performed by experts optimizing the automatic solutions given by the former implementation. In addition, we introduced a criterion for selecting a variable number of wavelet coefficients, which allowed discriminating more units but keeping a low number of features when a small number of units was present in the recording or when the background noise was high. In fact, using all the coefficients under low signal-to-noise ratios poses a problem for clustering methods in high-dimensional spaces, which is associated with the “curse of dimensionality” (Bishop 2006). Importantly, the improved performance achieved by the new dimensionality reduction was done with an effective implementation that led to computing times that where smaller than those of other algorithms.

Using data from real single-channel recordings, the new algorithm successfully detected, in a fully unsupervised way, ~95% of the clusters obtained by expert sorters, who manually optimized the solution of the former *Wave_clus* implementation. This was accomplished while maintaining a low number of false positives. When tested with single-channel simulated data, the new implementation with 10 wavelet coefficients achieved the same performance as the one from the expert sorters, showing that the modifications introduced accomplished a successful automatization. However, when the variable number of wavelet coefficients was used, the proposed algorithm was able to correctly detect more than the eight neurons identified by expert sorters using the former *Wave_clus* (while maintaining a low number of false positives). The performance with the new *Wave_clus* implementation was also significantly better than the one obtained with the algorithms Klusta and Combinato.

A good performance of the proposed algorithm was also observed with real and simulated data from tetrode recordings and was significantly better than the one obtained with several recently introduced algorithms designed for multichannel recordings: Klusta, MountainSort, Kilosort, and SpyKING CIRCUS. In addition, the implementation of *Wave_clus* is modular, in the sense that the feature extraction and clustering steps are decoupled. Therefore, it is possible, for example, to maintain the proposed method to select wavelet coefficients, while replacing SPC with a different clustering algorithm, such as the ones used by MountainSort (Chung et al. 2017) or SpyKING CIRCUS (Rodriguez and Laio 2014). Alternatively, a different method for feature extraction might be introduced while maintaining the SPC and the steps introduced here to provide a fully automatic solution.

Although the new implementation of *Wave_clus* showed good performance with real data, there is still room for improvement. When one performs long-term recordings, the stability of the spike waveforms can be affected by different causes, such as electrode drifts or changes in the recording conditions. Different solutions have been proposed to tackle this issue (Rey et al. 2015b). In fact, Combinato (Niediek et al. 2016) offers a novel approach to reliably track neurons over long periods of time, and it is a subject of further investigation to develop and compare different methodologies to optimally track neurons over days. Another problem observed in real data is due to overlapping spikes (Ekanadham et al. 2014; Franke et al. 2015; Rey et al. 2015b). This issue is normally diminished by simultaneously recording from different channels located close to each other (so that the overlap is not seen in all of them). The new *Wave_clus* implementation could be used in this context, although further research is required to find a good way of grouping the information from different channels and then properly combining the results. Finally, quality metrics can be incorporated to the sorting algorithm (Harris et al. 2016; Joshua et al. 2007; Rey et al. 2015b), and spatial preprocessing can be considered for large electrode arrays (Huang and Miller 2005; Musial et al. 2002).

The development of automatic and reliable spike-sorting algorithms is becoming critical, given that, within the next 10 years, we will likely witness the number of recording sites going up to thousands (Alivisatos et al. 2013; Stevenson and Kording 2011). The amount and complexity of the data to be produced by the next generation of probes are too large to be handled by researchers in a supervised way. In this context, the only viable option to fully take advantage of technological developments is to accompany them with the development of easy-to-use and properly validated tools for fully automatic spike sorting (Einevoll et al. 2012; Harris et al. 2016). In this respect, we have here presented a fully automatic algorithm that, not only matched, but also outperformed the performance of supervised algorithms and outperformed the performance of previously proposed unsupervised implementations.

## GRANTS

This research was supported by the Medical Research Council (G1002100) and the Human Frontiers Research Program.

## DISCLOSURES

No conflicts of interest, financial or otherwise, are declared by the authors.

## AUTHOR CONTRIBUTIONS

F.C. and H.G.R. analyzed data; F.C., H.G.R., and R.Q.Q. interpreted results of experiments; F.C. and H.G.R. prepared figures; F.C. and H.G.R. drafted manuscript; F.C., H.G.R., and R.Q.Q. edited and revised manuscript; F.C., H.G.R., and R.Q.Q. approved final version of manuscript; H.G.R. and R.Q.Q. conceived and designed research; R.Q.Q. performed experiments.
